# Supplementary material for: Influenza-associated hospitalisation and mortality rates among global Indigenous populations; a systematic review and meta-analysis
Source: PLOS Glob Public Health. 2023 Apr 13;3(4):e0001294. doi: 10.1371/journal.pgph.0001294 (PMC10101428; doi:10.1371/journal.pgph.0001294)
Supplement: S1 Text — Table A: Search StrategyTable B: List of excluded studies (from full text review)Table C: Features of included studiesTable D: Notes about hospitalisation- and mortality-rate calculationsTable E: Quality assessment of included studiesTable F: JBI risk of bias assessment–comments.References. (DOC) [file pgph.0001294.s002.doc]

# Supplementary Material: Influenza-associated hospitalisation and mortality rates among global Indigenous populations; a systematic review and meta-analysis

*Juliana M. Betts, Aaron L. Weinman, Jane Oliver, Maxwell Braddick, Siyu Huang, Matthew Nguyen, Adrian Miller, Steven Y.C. Tong, Katherine B. Gibney*

# Table A: Search Strategy

| **Database** | **Search Strategy 2017** | **Updated search July 2021 (search terms 2017 with the following limitations)** |
| --- | --- | --- |
| Medline OVID | (Indigenous or Aborigin* or native or trib* or First nation* or Maori or Inuit* or Indians, North American or Torres Strait Island* or Dai or Tibet* or Mon or Sherpa or Rai or Magar or Tamang or FATA or Sami or Nenet* or Baka or Pygm* or Maasai or Ijaw or Fulani or Metis or Mapuche or Kuna Yala or Embera Wounaan or Ngabe Bugle).mp. [mp=title, abstract, original title, name of substance word, subject heading word, floating sub-heading word, keyword heading word, organism supplementary concept word, protocol supplementary concept word, rare disease supplementary concept word, unique identifier, synonyms]  AND  Influenza.mp. [mp=title, abstract, original title, name of substance word, subject heading word, floating sub-heading word, keyword heading word, organism supplementary concept word, protocol supplementary concept word, rare disease supplementary concept word, unique identifier, synonyms]  AND  (hospital* or mortality or death or fatal*).mp. [mp=title, abstract, original title, name of substance word, subject heading word, floating sub-heading word, keyword heading word, organism supplementary concept word, protocol supplementary concept word, rare disease supplementary concept word, unique identifier, synonyms] | LIMIT search to 2017-12 July 2021 and ENGLISH language, hand search and remove anything on Medline before 13 June 2017. |
| Pubmed | ((Indigenous[Title/Abstract] OR Aborigin*[Title/Abstract] OR native[Title/Abstract] OR trib*[Title/Abstract] OR First nation*[Title/Abstract] OR Maori[Title/Abstract] OR Inuit*[Title/Abstract] OR Indians, North American[Title/Abstract] OR Torres Strait Island*[Title/Abstract] OR Dai[Title/Abstract] OR Tibet*[Title/Abstract] OR Mon[Title/Abstract] OR Sherpa[Title/Abstract] OR Rai[Title/Abstract] OR Magar[Title/Abstract] OR Tamang[Title/Abstract] OR FATA[Title/Abstract] OR Sami[Title/Abstract] OR Nenet*[Title/Abstract] OR Baka[Title/Abstract] OR Pygm*[Title/Abstract] OR Maasai[Title/Abstract] OR Ijaw[Title/Abstract] OR Fulani[Title/Abstract] OR Metis[Title/Abstract] OR Mapuche[Title/Abstract] OR Kuna Yala[Title/Abstract] OR Embera Wounaan[Title/Abstract] OR Ngabe Bugle[Title/Abstract]) AND (Influenza[Title/Abstract])) AND (hospital*[Title/Abstract] OR mortality[Title/Abstract] OR death[Title/Abstract] OR fatal*[Title/Abstract]) | Limit to English and 13 Jun 2017 to 12 July 2021 |
| Embase | (Indigenous or Aborigin* or native or trib* or First nation* or Maori or Inuit* or Indians, North American or Torres Strait Island* or Dai or Tibet* or Mon or Sherpa or Rai or Magar or Tamang or FATA or Sami or Nenet* or Baka or Pygm* or Maasai or Ijaw or Fulani or Metis or Mapuche or Kuna Yala or Embera Wounaan or Ngabe Bugle).mp. [mp=title, abstract, heading word, drug trade name, original title, device manufacturer, drug manufacturer, device trade name, keyword, floating subheading word, candidate term word]  AND  Influenza.mp. [mp=title, abstract, heading word, drug trade name, original title, device manufacturer, drug manufacturer, device trade name, keyword, floating subheading word, candidate term word]  AND  (hospital* or mortality or death or fatal*).mp. [mp=title, abstract, original title, name of substance word, subject heading word, floating sub-heading word, keyword heading word, organism supplementary concept word, protocol supplementary concept word, rare disease supplementary concept word, unique identifier, synonyms] | LIMIT search to 2017-12 July 2021 and ENGLISH language, hand search and remove anything on Medline before 13 June 2017. |
| Cochrane Central Register of Controlled Trials | (Indigenous or Aborigin* or native or trib* or First nation* or Maori or Inuit* or Indians, North American or Torres Strait Island* or Dai or Tibet* or Mon or Sherpa or Rai or Magar or Tamang or FATA or Sami or Nenet* or Baka or Pygm* or Maasai or Ijaw or Fulani or Metis or Mapuche or Kuna Yala or Embera Wounaan or Ngabe Bugle) as Title, abstract, keyword.  AND  (Influenza) as Title, abstract, keyword.  AND  (hospital* or mortality or death or fatal*) as Title, abstract, keyword. | With Publication Year from 2017 to 2021, in Trials. |
| CINAHL Complete | (Indigenous or Aborigin* or native or trib* or First nation* or Maori or Inuit* or Indians, North American or Torres Strait Island* or Dai or Tibet* or Mon or Sherpa or Rai or Magar or Tamang or FATA or Sami or Nenet* or Baka or Pygm* or Maasai or Ijaw or Fulani or Metis or Mapuche or Kuna Yala or Embera Wounaan or Ngabe Bugle)  AND  influenza  AND  (hospital* or mortality or death or fatal*) | No search field selected, English language, results limited to published between June 2017 and 13 July 2021. |

# Table B: List of excluded studies (from full text review)

|  | **Citation** | **Reason for Exclusion** |
| --- | --- | --- |
|  | Gai JF, Ji Y, Yao JH. Acquired immunocompromise in children with serious influenza A (H1N1). Zhongguo Dang dai er ke za zhi= Chinese Journal of Contemporary Pediatrics. 2010 Oct 1;12(10):829-30. | Not in English |
|  | Bhat N, Tokarz R, Jain K, Haq S, Weatherholtz R, Chandran A, Karron R, Reid R, Santosham M, O’Brien KL, Lipkin WI. A prospective study of agents associated with acute respiratory infection among young American Indian children. The Pediatric infectious disease journal. 2013 Aug;32(8):e324. | Wrong comparator |
|  | Mahoney MC, Michalek AM. The Health Status of American Indians/Alaska Natives: General Patterns of Mortality. Familyl Medicine – Kansas City 1998 Mar 1;30:190-5. | Wrong comparator |
|  | Andrews MM, Krouse SA. Research on excess deaths among American Indians and Alaska Natives: a critical review. Journal of cultural diversity. 1995 Jan 1;2(1):8-15. | Wrong outcomes |
|  | Appuhamy RD, Beard FH, Phung HN, Selvey CE, Birrell FA, Culleton TH. The changing phases of pandemic (H1N1) 2009 in Queensland: an overview of public health actions and epidemiology. Medical journal of Australia. 2010 Jan;192(2):94-7. | Wrong outcomes |
|  | Arias E, Anderson RN, Kung HC, Murphy SL, Kochanek KD. Deaths: final data for 2001. National vital statistics reports. 2003 Sep 18;52(3):1-16. | Wrong outcomes |
|  | Bagshaw SM, Sood MM, Long J, Fowler RA, Adhikari NK. Acute kidney injury among critically ill patients with pandemic H1N1 influenza A in Canada: cohort study. BMC nephrology. 2013 Dec;14(1):1-1. | Wrong outcomes |
|  | Banerji A, Bell A, Mills EL, McDonald J, Subbarao K, Stark G, Eynon N, Loo VG. Lower respiratory tract infections in Inuit infants on Baffin Island. Cmaj. 2001 Jun 26;164(13):1847-50. | Wrong outcomes |
| 1. # | Bissielo A, Huang S, Baker M, Beasley R. Seroprevalence of the 2009 influenza A (H1N1) pandemic in New Zealand. | Wrong outcomes |
|  | Blyth CC, Jacoby P, Effler PV, Kelly H, Smith DW, Borland ML, Willis GA, Levy A, Keil AD, Richmond PC. Influenza vaccine effectiveness and uptake in children at risk of severe disease. The Pediatric Infectious Disease Journal. 2016 Mar 1;35(3):309-15. | Wrong outcomes |
|  | Blyth CC, Jacoby P, Effler PV, Kelly H, Smith DW, Robins C, Willis GA, Levy A, Keil AD, Richmond PC. Effectiveness of trivalent flu vaccine in healthy young children. Pediatrics. 2014 May;133(5):e1218-25. | Wrong outcomes |
|  | Cano E, Suarez N, Freire A. Deaths From Respiratory Infections in Ecuadorian Indigenous and Nonindigenous People. Chest. 2015 Oct 1;148(4):129A. | Wrong outcomes |
| 1. # | Centres for Disease Control (Ritger KA, Jones RC, Weaver KN, Ramirez E, Smith S, Morita JY, Lohff CJ, Black SB, Jones JD, Wong W, Samala U). 2009 Pandemic influenza A (H1N1) virus infections-Chicago, Illinois, April-July 2009. Morbidity and Mortality Weekly Report. 2009;58(33):913-8. | Wrong outcomes |
|  | Chang AB, Chang CC, O'Grady K, Torzillo PJ. Lower respiratory tract infections. Pediatric Clinics. 2009 Dec 1;56(6):1303-21. | Wrong outcomes |
|  | Chang MH, Moonesinghe R, Athar HM, Truman BI. Trends in disparity by sex and race/ethnicity for the leading causes of death in the United States—1999-2010. Journal of Public Health Management and Practice. 2016 Jan 1;22:S13-24. | Wrong outcomes |
|  | Charland KM, Brownstein JS, Verma A, Brewer T, Jones S, Hoen AG, Buckeridge DL. Increased influenza-related healthcare utilization by residents of an urban aboriginal community. Epidemiology & Infection. 2011 Dec;139(12):1902-8. | Wrong outcomes |
|  | Chen JM, Chen JW, Dai JJ, Sun YX. A survey of human cases of H5N1 avian influenza reported by the WHO before June 2006 for infection control. American journal of infection control. 2007 Sep 1;35(7):467-9. | Wrong outcomes |
|  | Cheng AC, Kotsimbos T, Reynolds A, Bowler SD, Brown SG, Hancox RJ, Holmes M, Irving L, Jenkins C, Thompson P, Simpson G. Clinical and epidemiological profile of patients with severe H1N1/09 pandemic influenza in Australia and New Zealand: an observational cohort study. BMJ open. 2011 Jan 1;1(1):e000100. | Wrong outcomes |
|  | Christensen M, Kightlinger L. Premature mortality patterns among American Indians in South Dakota, 2000–2010. American journal of preventive medicine. 2013 May 1;44(5):465-71. | Wrong outcomes |
|  | Cooper, M.J. Risk factors for severe outcomes and impact of vaccination on pneumonia and influenza within active US military populations 2000-2012. American Journal of Tropical Medicine and Hygeine. 2014 November 1:16. | Wrong outcomes |
|  | Crighton EJ, Elliott SJ, Moineddin R, Kanaroglou P, Upshur R. A spatial analysis of the determinants of pneumonia and influenza hospitalizations in Ontario (1992–2001). Social science & medicine. 2007 Apr 1;64(8):1636-50. | Wrong outcomes |
|  | Das RR, Sami A, Lodha R, Jain R, Broor S, Kaushik S, Singh BB, Ahmed M, Seth R, Kabra SK. Clinical profile and outcome of swine flu in Indian children. Indian pediatrics. 2011 May;48(5):373-8. | Wrong outcomes |
|  | Day GE, Provost E, Lanier AP. Alaska native mortality rates and trends. Public health reports. 2009 Jan;124(1):54-64. | Wrong outcomes |
|  | Decker JF. Depopulation of the Northern Plains natives. Social science & medicine. 1991 Jan 1;33(4):381-93. | Wrong outcomes |
|  | Drewette-Card RJ, Landen MG. The disparity change score: a new methodology to examine health disparities in New Mexico. Journal of public health management and practice. 2005 Nov 1;11(6):484-92. | Wrong outcomes |
|  | Eick AA, Uyeki TM, Klimov A, Hall H, Reid R, Santosham M, O’Brien KL. Maternal influenza vaccination and effect on influenza virus infection in young infants. Archives of pediatrics & adolescent medicine. 2011 Feb 7;165(2):104-11. | Wrong outcomes |
|  | Gracey M, Sun W, Somerford P. Changing mortality patterns in Kimberley Aborigines. Internal medicine journal. 2015 Sep;45(9):905-8. | Wrong outcomes |
|  | Greer AL, Tuite A, Fisman DN. Age, influenza pandemics and disease dynamics. Epidemiology & Infection. 2010 Nov;138(11):1542-9. | Wrong outcomes |
|  | Hadler JL, Yousey-Hindes K, Pérez A, Anderson EJ, Bargsten M, Bohm SR, Hill M, Hogan B, Laidler M, Lindegren ML, Lung KL. Influenza-related hospitalizations and poverty levels—United States, 2010–2012. Morbidity and Mortality Weekly Report. 2016 Feb 12;65(5):101-5. | Wrong outcomes |
|  | Hennessy TW, Ritter T, Holman RC, Bruden DL, Yorita KL, Bulkow L, Cheek JE, Singleton RJ, Smith J. The relationship between in-home water service and the risk of respiratory tract, skin, and gastrointestinal tract infections among rural Alaska natives. American journal of public health. 2008 Nov;98(11):2072-8. | Wrong outcomes |
|  | Herceg A, Sharp PG, Arthur CG, Tongs JA. Pandemic (H1N1) 2009 influenza in an urban Aboriginal medical service. The Medical Journal of Australia. 2010 May 17;192(10):623. | Wrong outcomes |
|  | Hertz T, Oshansky CM, Roddam PL, DeVincenzo JP, Caniza MA, Jojic N, Mallal S, Phillips E, James I, Halloran ME, Thomas PG. HLA targeting efficiency correlates with human T-cell response magnitude and with mortality from influenza A infection. Proceedings of the National Academy of Sciences. 2013 Aug 13;110(33):13492-7. | Wrong outcomes |
|  | Holck P, Ehrsam Day G, Provost E. Mortality trends among Alaska Native people: successes and challenges. International journal of circumpolar health. 2013 Jan 31;72(1):21185. | Wrong outcomes |
|  | Hoyert DL, Mathews TJ, Menacker F, Strobino DM, Guyer B. Annual summary of vital statistics: 2004. Pediatrics. 2006 Jan;117(1):168-83. | Wrong outcomes |
|  | Jouvet P, Hutchison J, Pinto R, Menon K, Rodin R, Choong K, Kesselman M, Veroukis S, Dugas MA, Santschi M, Guerguerian AM. Critical illness in children with influenza A/pH1N1 2009 infection in Canada. Pediatric Critical Care Medicine. 2010 Sep 1;11(5):603-9. | Wrong outcomes |
|  | Knight M, Pierce M, Seppelt I, Kurinczuk JJ, Spark P, Brocklehurst P, McLintock C, Sullivan E, UK’s Obstetric Surveillance System, the ANZIC Influenza Investigators, and the Australasian Maternity Outcomes Surveillance System. Critical illness with AH1N1v influenza in pregnancy: a comparison of two population‐based cohorts. BJOG: An International Journal of Obstetrics & Gynaecology. 2011 Jan;118(2):232-9. | Wrong outcomes |
|  | Kochanek KD, Murphy SL, Anderson RN, Scott C. Deaths: final data for 2002. | Wrong outcomes |
|  | Kumar A, Zarychanski R, Pinto R, Cook DJ, Marshall J, Lacroix J, Stelfox T, Bagshaw S, Choong K, Lamontagne F, Turgeon AF. Critically ill patients with 2009 influenza A (H1N1) infection in Canada. Jama. 2009 Nov 4;302(17):1872-9. | Wrong outcomes |
|  | La Ruche G, Tarantola A, Barboza P, Vaillant L, Gueguen J, Gastellu-Etchegorry M. The 2009 pandemic H1N1 influenza and indigenous populations of the Americas and the Pacific. Eurosurveillance. 2009 Oct 22;14(42):19366. | Wrong outcomes |
|  | Menzies R, McIntyre P. Vaccine preventable diseases and vaccination policy for indigenous populations. Epidemiologic reviews. 2006 Aug 1;28(1):71-80. | Wrong outcomes |
|  | Moore HC, De Klerk N, Richmond P, Keil AD, Lindsay K, Plant A, Lehmann D. Seasonality of respiratory viral identification varies with age and Aboriginality in metropolitan Western Australia. The Pediatric infectious disease journal. 2009 Jul 1;28(7):598-603. | Wrong outcomes |
| 1. # | Pandemic Influenza Mortality and Morbidity Review Group. Report for the Minister of Health. New Zealand Ministry of Health. 2010 | Wrong outcomes |
|  | Pierse N, Kelly H, Thompson MG, Bissielo A, Radke S, Huang QS, Baker MG, Turner N. Influenza vaccine effectiveness for hospital and community patients using control groups with and without non-influenza respiratory viruses detected, Auckland, New Zealand 2014. Vaccine. 2016 Jan 20;34(4):503-9. | Wrong outcomes |
|  | Pollock SL, Sagan M, Oakley L, Fontaine J, Poffenroth L. Investigation of a pandemic H1N1 influenza outbreak in a remote First Nations community in northern Manitoba, 2009. Canadian journal of public health. 2012 Mar;103(2):90-3. | Wrong outcomes |
|  | Rhoades ER. The Major Respiratory Diseases of American Indians1-4. Am Rev Respir Dis. 1990;141:595-800. | Wrong outcomes |
|  | Samet JM, Key CR, Kutvirt DM, Wiggins CL. Respiratory disease mortality in New Mexico's American Indians and Hispanics. American Journal of Public Health. 1980 May;70(5):492-7. | Wrong outcomes |
|  | Shiels MS, Chernyavskiy P, Anderson WF, Best AF, Haozous EA, Hartge P, Rosenberg PS, Thomas D, Freedman ND, de Gonzalez AB. Trends in premature mortality in the USA by sex, race, and ethnicity from 1999 to 2014: an analysis of death certificate data. The Lancet. 2017 Mar 11;389(10073):1043-54. | Wrong outcomes |
|  | Singleton RJ, Bulkow LR, Miernyk K, DeByle C, Pruitt L, Hummel KB, Bruden D, Englund JA, Anderson LJ, Lucher L, Holman RC. Viral respiratory infections in hospitalized and community control children in Alaska. Journal of medical virology. 2010 Jul;82(7):1282-90. | Wrong outcomes |
|  | Suryaprasad A, Redd JT, Hancock K, Branch A, Steward‐Clark E, Katz JM, Influenza Serology Working Group, Fry AM, Cheek JE, American Indian and Alaska Native Pandemic Influenza A (H1N1) Investigation Team. Severe acute respiratory infections caused by 2009 pandemic influenza A (H 1 N 1) among A merican I ndians—southwestern U nited S tates, M ay 1–J uly 21, 2009. Influenza and other respiratory viruses. 2013 Nov;7(6):1361-9. | Wrong outcomes |
|  | Tomashek KM, Qin C, Hsia J, Iyasu S, Barfield WD, Flowers LM. Infant mortality trends and differences between American Indian/Alaska Native infants and white infants in the United States, 1989–1991 and 1998–2000. American Journal of Public Health. 2006 Dec;96(12):2222-7. | Wrong outcomes |
|  | Trenholme AA, Best EJ, Vogel AM, Stewart JM, Miller CJ, Lennon DR. Respiratory virus detection during hospitalisation for lower respiratory tract infection in children under 2 years in South Auckland, New Zealand. Journal of Paediatrics and Child Health. 2017 Jun;53(6):551-5. | Wrong outcomes |
|  | Wang C, Yu H, Horby PW, Cao B, Wu P, Yang S, Gao H, Li H, Tsang TK, Liao Q, Gao Z. Comparison of patients hospitalized with influenza A subtypes H7N9, H5N1, and 2009 pandemic H1N1. Clinical infectious diseases. 2014 Apr 15;58(8):1095-103. | Wrong outcomes |
|  | Webster D, Weerasinghe S, Stevens P. Morbidity and mortality rates in a Nova Scotia First Nations community, 1996–1999. Canadian journal of public health. 2004 Sep;95(5):369-74. | Wrong outcomes |
|  | Wilkinson K, Mitchell R, Taylor G, Amihod B, Frenette C, Gravel D, McGeer A, Suh KN, Wong A, Canadian Nosocomial Infection Surveillance Program. Laboratory-confirmed pandemic H1N1 influenza in hospitalized adults: findings from the Canadian Nosocomial Infections Surveillance Program, 2009-2010. Infection Control & Hospital Epidemiology. 2012 Oct;33(10):1043-6. | Wrong outcomes |
|  | Wilson DM, Truman CD. Long-term-care residents. Canadian Journal of Public Health. 2004 Sep;95(5):382-6. | Wrong outcomes |
|  | Wong CA, Gachupin FC, Holman RC, MacDorman MF, Cheek JE, Holve S, Singleton RJ. American Indian and Alaska native infant and pediatric mortality, United States, 1999–2009. American journal of public health. 2014 Jun;104(S3):S320-8. | Wrong outcomes |
|  | Zhou M, Wang H, Zhu J, Chen W, Wang L, Liu S, Li Y, Wang L, Liu Y, Yin P, Liu J. Cause-specific mortality for 240 causes in China during 1990–2013: a systematic subnational analysis for the Global Burden of Disease Study 2013. The Lancet. 2016 Jan 16;387(10015):251-72. | Wrong outcomes |
| 1. # | Jackson Pulver L, ANZIC Influenza Investigators. Critical illness due to 2009 A/H1N1 influenza in pregnant and postpartum women: population based cohort study. BMJ: British Medical Journal. 2010. | Wrong outcomes |
|  | Cunningham J, Condon JR. Premature mortality in Aboriginal adults in the Northern Territory, 1979‐1991. Medical Journal of Australia. 1996 Sep;165(6):309-12. | Wrong outcomes |
|  | Osama SM, Krishnamurti S, Rai J, Gupta DN. Outbreak of influenza A/Victoria/3/75 infection in a probationer nurses school. | Wrong outcomes |
|  | Singleton R, Karron RA, Kruse DG, Harrison LH, DeSmet IJ, Davidson NM, Petersen KM. RSV-associated hospitalizations in Alaska Native infants. International journal of circumpolar health. 1998 Jan 1;57:255-9. | Wrong outcomes |
|  | Blyth CC, Macartney KK, Hewagama S, Senenayake S, Friedman ND, Simpson G, Upham J, Kotsimbos T, Kelly P, Cheng AC. Influenza epidemiology, vaccine coverage and vaccine effectiveness in children admitted to sentinel Australian hospitals in 2014: the Influenza Complications Alert Network (FluCAN). Eurosurveillance. 2016 Jul 28;21(30):30301. | Wrong outcomes |
|  | Kool JL, Pavlin BI, Musto J, Dawainavesi A. Influenza surveillance in the Pacific Island countries and territories during the 2009 pandemic: an observational study. BMC Infectious Diseases. 2013 Dec;13(1):1-8. | Wrong patient population |
|  | Lafond KE, Nair H, Rasooly MH, Valente F, Booy R, Rahman M, Kitsutani P, Yu H, Guzman G, Coulibaly D, Armero J. Global role and burden of influenza in pediatric respiratory hospitalizations, 1982–2012: a systematic analysis. PLoS medicine. 2016 Mar 24;13(3):e1001977. | Wrong patient population |
| 1. # | Lindstrom S, Garten R, Balish A, Shu B, Emery S, Berman L, Barnes N, Sleeman K, Gubareva L, Villanueva J, Klimov A. Human infections with novel reassortant influenza A (H3N2) v viruses, United States, 2011. Emerging infectious diseases. 2012 May;18(5):834. | Wrong patient population |
|  | Mmbaga VM, Mwasekaga MJ, Mmbuji P, Matonya M, Mwafulango A, Moshi S, Emukule G, Katz MA. Results from the first 30 months of national sentinel surveillance for influenza in Tanzania, 2008–2010. The Journal of infectious diseases. 2012 Dec 15;206(suppl_1):S80-6. | Wrong patient population |
|  | Cheng A, Dwyer D, Holmes M, Irving L, Brown SG, Waterer G, Korman TM, Hunter C, Hewagama S, Friedman ND, Senanayake S. Influenza epidemiology, vaccine coverage and vaccine effectiveness in sentinel Australian hospitals in 2013: the Influenza Complications Alert Network. | Wrong setting |
|  | Cheng AC, Holmes M, Dwyer DE, Irving LB, Korman TM, Senenayake S, Macartney KK, Blyth CC, Brown S, Waterer G, Hewer R. Influenza epidemiology in patients admitted to sentinel Australian hospitals in 2015: the Influenza Complications Alert Network. Communicable Diseases Intelligence Quarterly Report. 2016;40(4):E521-6. | Wrong setting |
|  | Cheng AC, Holmes M, Senenayake S, Dwyer DE, Hewagama S, Korman T, Irving L, Brown S, Waterer G, Hunter C, Friedman ND. Influenza epidemiology in adults admitted to sentinel Australian hospitals in 2014: the Influenza Complications Alert Network (FluCAN). Communicable Diseases Intelligence Quarterly Report. 2015 Sep 30;39(3):E355-60. | Wrong setting |
|  | Kelly PM, Kotsimbos T, Reynolds A, Wood‐Baker R, Hancox B, Brown SG, Holmes M, Simpson G, Bowler S, Waterer G, Irving LB. FluCAN 2009: initial results from sentinel surveillance for adult influenza and pneumonia in eight Australian hospitals. Medical Journal of Australia. 2011 Feb;194(4):169-74. | Wrong setting |
|  | Cheng AC, Brown S, Waterer G, Holmes M, Senenayake S, Friedman ND, Hewagama S, Simpson G, Wark P, Upham J, Korman T. Influenza epidemiology, vaccine coverage and vaccine effectiveness in sentinel Australian hospitals in 2012: the Influenza Complications Alert Network (FluCAN). Communicable Diseases Intelligence Quarterly Report. 2013 Sep 30;37(3):E246-52. | Wrong setting |
|  | Bertozzi S, Kelso A, Tashiro M, Savy V, Farrar J, Osterholm M, Jameel S, Muller CP. Pandemic flu: from the front lines. Interviewed by Declan Butler. Nature. 2009 Sep 1;461(7260):20-1. | Wrong study design |
|  | Poster Summaries, 9th Canadian Immunization Conference. Can J Infect Dis Med Microbiol. 2010;21(4):173. | Wrong study design |
|  | The Lancet. Poor health outcomes in Native Americans and Alaska Natives. The Lancet. 2014;383(9928):1522. | Wrong study design |
|  | Barnett H, Fields J, Milles G, SILVERSTEIN J, Bernstein A. Medical conditions in Alaska: A report by a group sent by the American Medical Association. Journal of the American Medical Association. 1947 Oct 25;135(8):500-10. | Wrong study design |
|  | Bierdrager J, De Rook H. Health conditions in Netherlands New Guinea. Documenta de Medicina Geographica et Tropica. 1954;6:252-66. | Wrong study design |
|  | Charania NA, Tsuji LJ. The 2009 H1N1 pandemic response in remote First Nation communities of Subarctic Ontario: barriers and improvements from a health care services perspective. International Journal of Circumpolar Health. 2011 Feb 18;70(5):564-75. | Wrong study design |
|  | Duvvuri VR, Duvvuri B, Alice C, Wu GE, Gubbay JB, Wu J. Preexisting CD4+ T-cell immunity in human population to avian influenza H7N9 virus: whole proteome-wide immunoinformatics analyses. PloS one. 2014 Mar 7;9(3):e91273. | Wrong study design |
|  | Ewart WB. Causes of mortality in a subarctic settlement (York Factory, Man.), 1714-1946. Canadian Medical Association Journal. 1983 Sep 9;129(6):571. | Wrong study design |
|  | Ferreira MU, Castro MC. No longer a deadly encounter?. Pathogens and global health. 2015 Oct 3;109(7):307-8. | Wrong study design |
|  | Fléchelles O, Fowler R, Jouvet P. H1N1 pandemic: clinical and epidemiologic characteristics of the Canadian pediatric outbreak. Expert Review of Anti-infective Therapy. 2013 Jun 1;11(6):555-63. | Wrong study design |
|  | Ganley ML. The dispersal of the 1918 influenza virus on the Seward Peninsula, Alaska: An ethnohistoric reconstruction. International Journal of Circumpolar Health. 1998 Jan 1;57:247-51. | Wrong study design |
|  | Grant EJ, Quiñones-Parra SM, Clemens EB, Kedzierska K. Human influenza viruses and CD8+ T cell responses. Current opinion in virology. 2016 Feb 1;16:132-42. | Wrong study design |
|  | Green ME, Wong ST, Lavoie JG, Kwong J, MacWilliam L, Peterson S, Liu G, Katz A. Admission to hospital for pneumonia and influenza attributable to 2009 pandemic A/H1N1 influenza in First Nations communities in three provinces of Canada. BMC Public Health. 2013 Dec;13(1):1-8. | Wrong study design |
|  | Groom AV, Jim C, LaRoque M, Mason C, McLaughlin J, Neel L, Powell T, Weiser T, Bryan RT. Pandemic influenza preparedness and vulnerable populations in tribal communities. American journal of public health. 2009 Oct;99(S2):S271-8. | Wrong study design |
|  | Ann Herring D, Sattenspiel L. Social contexts, syndemics, and infectious disease in northern Aboriginal populations. American Journal of Human Biology: The Official Journal of the Human Biology Association. 2007 Mar;19(2):190-202. | Wrong study design |
|  | Hirve S, Krishnan A, Dawood FS, Lele P, Saha S, Rai S, Gupta V, Lafond KE, Juvekar S, Potdar V, Broor S. Incidence of influenza-associated hospitalization in rural communities in western and northern India, 2010–2012: a multi-site population-based study. Journal of Infection. 2015 Feb 1;70(2):160-70. | Wrong study design |
| 1. # | Joseph, T., Menzies, R. McIntyre, P. Vaccination for our mob. 2006. Commonwealth of Australia Department of Health and Ageing. | Wrong study design |
|  | Khieu TQ, Pierse N, Telfar-Barnard LF, Huang QS, Baker MG. Estimating the contribution of influenza to hospitalisations in New Zealand from 1994 to 2008. Vaccine. 2015 Aug 7;33(33):4087-92. | Wrong study design |
|  | Khieu TQ, Pierse N, Telfar-Barnard LF, Zhang J, Huang QS, Baker MG. Modelled seasonal influenza mortality shows marked differences in risk by age, sex, ethnicity and socioeconomic position in New Zealand. Journal of Infection. 2017 Sep 1;75(3):225-33. | Wrong study design |
|  | Killingray D. The influenza pandemic of 1918–1919 in the British Caribbean. Social History of Medicine. 1994 Apr 1;7(1):59-87. | Wrong study design |
|  | Kumar S, Quinn SC. Existing health inequalities in India: informing preparedness planning for an influenza pandemic. Health Policy and Planning. 2012 Sep 1;27(6):516-26. | Wrong study design |
| 1. # | La D, Czarnecki C, El-Gabalawy H, Kumar A, Meyers AF, Bastien N, Simonsen JN, Plummer FA, Luo M. Enrichment of variations in KIR3DL1/S1 and KIR2DL2/L3 among H1N1/09 ICU patients: an exploratory study. PloS one. 2011 Dec 28;6(12):e29200. | Wrong study design |
|  | Lester-Smith D, Zurynski YA, Booy R, Festa MS, Kesson AM, Elliott EJ. The burden of childhood influenza in a tertiary paediatric setting The researchers in this study undertook a retrospective medical record review to investigate the impact of admissions of children with laboratory-confirmed influenza to The Children’s Hospital at Westmead, Sydney, during 2007. Page last updated: 18 September 2009. | Wrong study design |
|  | Mamelund SE. Geography may explain adult mortality from the 1918–20 influenza pandemic. Epidemics. 2011 Mar 1;3(1):46-60. | Wrong study design |
|  | McIntyre PB, Menzies RI. Immunisation: reducing health inequality for Indigenous Australians. The Medical Journal of Australia. 2005 Mar 7;182(5):207-8. | Wrong study design |
| 1. # | Menzies R, McIntyre P. Vaccine preventable diseases and vaccination policy for indigenous populations. Epidemiologic reviews. 2006 Aug 1;28(1):71-80. | Wrong study design |
|  | Miller A, Durrheim DN. Aboriginal and Torres Strait Islander communities forgotten in new Australian National Action Plan for Human Influenza Pandemic:" Ask us, listen to us, share with us". Medical Journal of Australia. 2010 Sep 20;193(6):316. | Wrong study design |
|  | Moore H, Burgner D, Carville K, Jacoby P, Richmond P, Lehmann D. Diverging trends for lower respiratory infections in non‐Aboriginal and Aboriginal children. Journal of paediatrics and child health. 2007 Jun;43(6):451-7. | Wrong study design |
|  | Morrison KT, Buckeridge DL, Xiao Y, Moghadas SM. The impact of geographical location of residence on disease outcomes among Canadian First Nations populations during the 2009 influenza A (H1N1) pandemic. Health & place. 2014 Mar 1;26:53-9. | Wrong study design |
|  | Nowlan M, Turner N, Kiedrzynski T, Jennings L. Synopsis of New Zealand's inaugural influenza symposium-influenza is a severe vaccine-preventable disease. The New Zealand Medical Journal (Online). 2015 Mar 13;128(1410):30. | Wrong study design |
|  | O'Grady KF, Torzillo PJ, Sloots T, Rockett R, Lambert SB. Surveillance for viral pathogens causing acute respiratory infections in remote indigenous communities in Australia: a comparison of sample transport methods. Paediatric Respiratory Reviews. 2010(11):S107. | Wrong study design |
|  | Pearce DC, McCaw JM, McVernon J, Mathews JD. Influenza as a trigger for cardiovascular disease: An investigation of serotype, subtype and geographic location. Environmental research. 2017 Jul 1;156:688-96. | Wrong study design |
|  | Penman-Aguilar A, Tucker MJ, Groom AV, Reilley BA, Klepacki S, Cullen T, Gebremariam C, Redd JT. Validation of algorithm to identify American Indian/Alaska Native pregnant women at risk from pandemic H1N1 influenza. American journal of obstetrics and gynecology. 2011 Jun 1;204(6):S46-53. | Wrong study design |
|  | Pool DI. The effects of the 1918 pandemic of influenza on the Maori population of New Zealand. Bulletin of the History of Medicine. 1973 May 1;47(3):273-81. | Wrong study design |
|  | Pritchard EN, Jutel A, Tollafield S. Positive provider interventions for enhancing influenza vaccination uptake among Pacific Peoples in New Zealand. NZ Med J. 2011 Nov 25;124(1346):75-82. | Wrong study design |
|  | Regan AK, Mak DB, Thomas T, Effler PV. Seasonal influenza vaccination in Aboriginal children in Western Australia in 2015. Australian Family Physician. 2016 Oct;45(10):726-7. | Wrong study design |
|  | Saito T, Uchida Y, Myint WW, Thein WZ, Watanabe C, Takemae N, Mase M, Okamatsu M, Mar A, Mon CC, Gawng LT. Characterisation of highly pathogenic avian influenza viruses in Myanmar. The Veterinary Record. 2008 Dec 13;163(24):722. | Wrong study design |
|  | Schaffer A, Muscatello D, Cretikos M, Gilmour R, Tobin S, Ward J. The impact of influenza A (H1N1) pdm09 compared with seasonal influenza on intensive care admissions in New South Wales, Australia, 2007 to 2010: a time series analysis. BMC Public Health. 2012 Dec;12(1):1-4. | Wrong study design |
|  | Singh SS, Muruganandam N, Chaaithanya IK, Bhattacharya D, Sugunan AP, Nayak AK, Roy A, Shriram AN, Vijayachari P. H1N1 influenza A outbreak among the Nicobarese, an aboriginal tribe of the Andaman and Nicobar Islands, India. public health. 2011 Aug 1;125(8):501. | Wrong study design |
|  | Summers JA, Shanks GD, Baker MG, Wilson N. Severe impact of the 1918–19 pandemic influenza in a national military force. NZ Med J. 2013 Jul 12;126(1378):36-47. | Wrong study design |
| 1. # | ANZIC Influenza Investigators. Critical care services and 2009 H1N1 influenza in Australia and New Zealand. New England Journal of Medicine. 2009 Nov 12;361(20):1925-34. | Wrong study design |
| 1. # | Trauer JM, Bandaranayake D, Booy R, Chen MI, Cretikos M, Dowse GK, Dwyer DE, Greenberg ME, Huang QS, Khandaker G, Kok J. Seroepidemiologic effects of influenza A (H1N1) pdm09 in Australia, New Zealand, and Singapore. Emerging infectious diseases. 2013 Jan;19(1):92. | Wrong study design |
|  | Underwood JH. Effects of the 1918 influenza pandemic mortality experience on subsequent fertility of the native population of Guam. Micronesica. 1984 Jan 1;19(1-2):1-0. | Wrong study design |
|  | Walker RS, Sattenspiel L, Hill KR. Mortality from contact-related epidemics among indigenous populations in Greater Amazonia. Scientific reports. 2015 Sep 10;5(1):1-9. | Wrong study design |
|  | Cunningham J, Condon JR. Premature mortality in Aboriginal adults in the Northern Territory, 1979‐1991. Medical Journal of Australia. 1996 Sep;165(6):309-12. | Wrong study design |
|  | Wilson N, Baker M. Ninety years on: What we still need to learn from" Black November" 1918 about pandemic influenza. The New Zealand Medical Journal (Online). 2008 Nov 7;121(1285). | Wrong study design |
|  | Wilson N, Barnard LT, Summers JA, Shanks GD, Baker MG. Differential mortality rates by ethnicity in 3 influenza pandemics over a century, New Zealand. Emerging infectious diseases. 2012 Jan;18(1):71. | Wrong study design |
|  | Wilson N, Oliver J, Rice G, Summers JA, Baker MG, Waller M, Shanks GD. Age-specific mortality during the 1918–19 influenza pandemic and possible relationship to the 1889–92 influenza pandemic. The Journal of infectious diseases. 2014 Sep 15;210(6):993-5. | Wrong study design |
|  | Zurynski, Y.; Booy, R.; Elliott, E. Influenza complications and deaths reported to the APSU among Aboriginal and Torres Strait Islander and non-Aboriginal and Torres Strait Islander children in 2008, 2009 and 2010. Journal of Paediatric Child Health. 2011 47;17. | Wrong study design |
|  | Zurynski, Y.; Lester-Smith, D.; Booy, R.; Festa, M.; Kesson, A.; Elliott, E. Influenza H1N1 2009 pandemic: Severe complications and deaths in children <15 years. J Paediatric Child Health. 2010, 46:16 | Wrong study design |
|  | Muschenh. C, Knight V, Cobb J, Riley H, Rabeau E, Dammin G, Mcdermot. W, Wilson M, Hook E, Shinefie. Hr, Johnson C. Forth National Conference on Indian Health. I. Respiratory Infections. Archives Of Environmental Health. 1968 Jan 1;17(2):247. | Wrong study design |
|  | Late News, Modern Healthcare 2009;39(37):4-4 | Wrong study design |
|  | Czarnecki, C. Analysis of HLA class i and class II antigens of ICU patients with severe response to H1N1 infection in the 2009 H1N1 pandemic. Journal of Antivirals and Antiretrovirals. Conference: International Conference and Exhibition on Virology | Wrong study design |
|  | Gu S, Long J, Menon K, Cook D, McGeer A, Kumar A, Jouvet P, Marshall J, Hutchison J, Fowler R, ICU-Flu Investigators OB. A Comparison Of The First And Second Waves Of H1N1-Related Critical Illness In Canada. InB49. Intensive Care Unit Management 2011 May (pp. A3141-A3141). American Thoracic Society. | Wrong study design |
|  | Henry, C.; Richter, D.; Richardson, R.; Whitall, B.; Curtis, G. Implementing pandemic influenza response in a first nation community in Alberta. Canadian Journal of Infectious Diseases and Medical Microbiology. 2010 Winter 21 (4); 174-175. | Wrong study design |
|  | Jung J, Fowler R, Long J, Zarychanski R, Rodin R, Cook DJ, Jouvet P, Marshall J, Kumar A, ICU-Flu Investigators OB. 2009-2010 H1N1-Related Critical Illness Among Aboriginal Canadians And Non-Aboriginal Canadians. InB49. Intensive Care Unit Management 2011 May (pp. A3140-A3140). American Thoracic Society. | Wrong study design |
|  | Kaposy C. Accounting for vulnerability to illness and social disadvantage in pandemic critical care triage. The Journal of clinical ethics. 2010 Jan 1;21(1):23-9. | Wrong study design |
|  | McNeil, S. A.; Johnstone, J.; Ambrose, A.; Loeb, M.; Russell, M.; Trottier, S.; Boivin, G.; McCarthy, A.; Henderson, E.; Stiver, G.; Halperin, S.; McGeer, A. Sentinel surveillance for influenza in Canadian hospitals: Experience of the public health agency of Canada (PHAC)/Canadian institutes of health research (CIHR) influenza research network (PCIRN) adult serious outcomes surveillance network. Canadian Journal of Infectious Diseases and Medical Microbiology 2010 (Winter) 21(4); 214. | Wrong study design |
|  | Rice G. Maori mortality in the 1918 influenza epidemic. New Zealand Population Review. 1983 Apr 1;9(1):44-61. | Wrong study design |
|  | Shanholtz, M. I. Impact of the 1967-68 influenza epidemic. Va Med Mon 1968 June 96 (6); 369-70. | Wrong study design |
|  | Silva DS, Nie JX, Rossiter K, Sahni S, Upshur RE. Contextualizing ethics: Ventilators, H1N1 and marginalized populations. | Wrong study design |
|  | Vachon, J.; Do, M. T.; Rodin, R.; Pulickal, J.; Pelletier, L. Impact of co-morbidities on outcome severity among pandemic H1N1 2009 (pH1N1) hospitalized cases in Canada. Canadian Journal of Infectious Diseases and Medical Microbiology. 2010 (Winter) 21(4): 219 | Wrong study design |
|  | Do, M. T.; Winchester, B.; Pelletier, L. Comparison of the age distribution among hospitalized pandemic H1N1 (pH1N1) cases during the first and second Waves in Canada. Canadian Journal of Infectious Diseases and Medical Microbiology. 2010 (Winter) 21(4): 219 | Wrong study design |
|  | Trenholme A, Lawrence S, Grant C, Prasad N, Newbern C, Todd A, Wood T, Huang S. Comparing respiratory virus burden among infants across emergency care and in-patient settings. European Respiratory Journal. Conference: European Respiratory Society Annual Congress (2016). 48. | Wrong study design |
| 1. # | Shalala DE, Trujillo MH, Hartz PE, Paisano EL. Trends in Indian health 1998–99. Washington, DC: United States Department Health and Human Services. 1999. | Wrong study design |
|  | Boggild AK, Yuan L, Low DE, McGeer AJ. The impact of influenza on the Canadian First Nations. Canadian Journal of Public Health. 2011 Sep;102(5):345-8. | Wrong study design |
|  | **Updated Search 2021** | |
|  | Prendergast C. Urgent air medical transfers for severe acute respiratory infections among children from northern Canada. Paediatrics and Child Health (Canada). 2019;24 (Supplement 2):e16. | Unable to access |
|  | Doxey M, Chrzaszcz L, Dominguez A, James RD. A Forgotten Danger: Burden of Influenza Mortality Among American Indians and Alaska Natives, 1999-2016. J Public Health Manag Pract. 2019;25 Suppl 5, Tribal Epidemiology Centers:Advancing Public Health in Indian Country for Over 20 Years:S7-S10. | Wrong Outcome |
|  | Probst J, Zahnd W, Breneman C. Declines in pediatric mortality fall short for rural us children. Health Affairs. 2019;38(12):2069-76. | Wrong Outcome |
|  | McHugh L, Andrews RM, Leckning B, Snelling T, Binks MJ. Baseline incidence of adverse birth outcomes and infant influenza and pertussis hospitalisations prior to the introduction of influenza and pertussis vaccination in pregnancy: a data linkage study of 78 382 mother-infant pairs, Northern Territory, Australia, 1994-2015. Epidemiol Infect. 2019;147:e233. | Wrong Outcome |
|  | Jung JJ, Pinto R, Zarychanski R, Cook DJ, Jouvet P, Marshall JC, et al. 2009-2010 Influenza A(H1N1)-related critical illness among Aboriginal and non-Aboriginal Canadians. PLoS ONE. 2017;12(10). | Wrong Outcome |
| 1. # | Yokomichi H, Mochizuki M, Lee JJ, Kojima R, Yokoyama T, Yamagata Z. Incidence of hospitalisation for severe complications of influenza virus infection in Japanese patients between 2012 and 2016: a cross-sectional study using routinely collected administrative data. BMJ Open. 2019; 9(1):e024687. https://doi.org/10.1136/bmjopen-2018-024687 PMID: 30782739 | Wrong Outcome |
| 1. # | Gravenstein S, Davidson HE, Taljaard M, Ogarek J, Gozalo P, Han L, et al. Comparative effectiveness of high-dose versus standard-dose influenza vaccination on numbers of US nursing home residents admitted to hospital: a cluster-randomised trial. Lancet Respir Med. 2017; 5(9):738–46. https://doi.org/ 10.1016/S2213-2600(17)30235-7 PMID: 28736045 | Wrong Outcome |
|  | Nolen LD, Seeman S, Desnoyers C, DeByle C, Klejka J, Bruden D, et al. Respiratory syncytial virus and influenza hospitalizations in Alaska native adults. J Clin Virol. 2020;127:104347. | Wrong population |
|  | Adisasmito W, Budayanti S, Aisyah DN, Coker R, Andayani AR, Smith GJD, et al. Surveillance and characterisation of influenza viruses among patients with influenza-like illness in Bali, Indonesia, July 2010-June 2014. BMC Infectious Diseases. 2019;19(1):N.PAG-N.PAG. | Wrong population |
|  | Biswal B, Dwibedi B, Hansa J, Kar SK. Bacterial and viral pathogen spectra of ARI among the children below 5 years age group in tribal and coastal regions of Odisha. Indian Journal of Public Health Research and Development. 2018;9(1):366-72. | Wrong population |
|  | Biswal B, Kar SK, Pal BB, Dwibedi B. Bacterial and viral etiology of acute respiratory illness among children from two different geographical localities of Odisha, 2015-2016. Journal of Pure and Applied Microbiology. 2018;12(2):993-1000. | Wrong population |
|  | Dawood FS, Chung JR, Kim SS, Zimmerman RK, Nowalk MP, Jackson ML, et al. Interim Estimates of 2019-20 Seasonal Influenza Vaccine Effectiveness - United States, February 2020. MMWR: Morbidity & Mortality Weekly Report. 2020;69(7):177-82. | Wrong study design |
|  | Khieu TQT, Pierse N, Telfar-Barnard LF, Zhang J, Huang QS, Baker MG. Modelled seasonal influenza mortality shows marked differences in risk by age, sex, ethnicity and socioeconomic position in New Zealand. J Infect. 2017;75(3):225-33. | Wrong study design |
|  | Trenholme AA, Best EJ, Vogel AM, Stewart JM, Miller CJ, Lennon DR. Respiratory virus detection during hospitalisation for lower respiratory tract infection in children under 2 years in South Auckland, New Zealand. J Paediatr Child Health. 2017;53(6):551-5. | Wrong study design |
|  | Sattenspiel L, Murray M, Mamelund SE. Co-circulating epidemics and health care access in early 20th century Alaska and Labrador: Implications for emerging diseases of the present. American Journal of Physical Anthropology. 2018;165 (Supplement 66):239. | Wrong study design |
|  | Thompson MG, Pierse N, Sue Huang Q, Prasad N, Duque J, Claire Newbern E, et al. Influenza vaccine effectiveness in preventing influenza-associated intensive care admissions and attenuating severe disease among adults in New Zealand 2012-2015. Vaccine. 2018;36(39):5916-25. | Wrong study design |
|  | Blyth CC, Cheng AC, Crawford NW, Clark JE, Buttery JP, Marshall HS, et al. The impact of new universal child influenza programs in Australia: Vaccine coverage, effectiveness and disease epidemiology in hospitalised children in 2018. Vaccine. 2020;38(13):2779-87. | Wrong study design |
|  | Blyth CC, Cheng AC, Macartney KK, McRae J, Clark JE, Marshall HS, et al. Influenza Epidemiology, Vaccine Coverage and Vaccine Effectiveness in Children Admitted to Sentinel Australian Hospitals in 2017: Results from the PAEDS-FluCAN Collaboration. Clin Infect Dis. 2019;68(6):940-8. | Wrong study design |
|  | Cheng AC, Holmes M, Dwyer DE, Irving L, Korman T, Senenayake S, et al. Influenza epidemiology in patients admitted to sentinel Australian hospitals in 2016: the Influenza Complications Alert Network (FluCAN). Commun Dis Intell Q Rep. 2017;41(4):E337-E47. | Wrong study design |
|  | Cheng AC, Holmes M, Dwyer DE, Senanayake S, Cooley L, Irving LB, et al. Influenza epidemiology in patients admitted to sentinel Australian hospitals in 2017: the Influenza Complications Alert Network (FluCAN). Commun Dis Intell (2018). 2019;43(09):16. | Wrong study design |
|  | Cheng AC, Holmes M, Dwyer DE, Senanayake S, Cooley L, Irving LB, et al. Influenza epidemiology in patients admitted to sentinel Australian hospitals in 2018: the Influenza Complications Alert Network (FluCAN). Commun Dis Intell (2018). 2019;43:18. | Wrong study design |
|  | Ao T, McCracken JP, Lopez MR, Bernart C, Chacon R, Moscoso F, et al. Hospitalization and death among patients with influenza, Guatemala, 2008-2012. BMC Public Health. 2019;19(Suppl 3):463. | Wrong study design |
| 1. # | GBD 2017 Influenza Collaborators. Mortality, morbidity, and hospitalisations due to influenza lower respiratory tract infections, 2017: an analysis for the Global Burden of Disease Study 2017. Lancet Respir Med. 2019; 7(1):69–89. https://doi.org/10.1016/S2213-2600(18)30496-X PMID: 30553848 | Wrong Study Design |

# indicates items identified through secondary search

# Table C: Features of included studies

| **Author, year** | **Study design** | **Setting** | **Age of participants** | **Indigenous Group** | **Benchmark Group** | **Time frame of study** | **Seasonal or pandemic** |
| --- | --- | --- | --- | --- | --- | --- | --- |
| Baker et al, 2009^1^ | Retrospective from surveillance data | New–Zealand (nation–wide) | All age groups | Maori | Europeans and others (excludes pacific peoples) | May – August 2009 | 2009 Influenza A (H1N1) pandemic |
| Bandaranayake et al, 2011^2^ | Retrospective from surveillance data | New–Zealand (nation–wide) | All age groups | Maori | Europeans and others (excludes pacific peoples) | January – October 2010 | 2009 Influenza A (H1N1) pandemic |
| Brooks et al, 2012^3^ | Retrospective cohort | U.S.A (New Mexico) | All age groups  Mean 44·2 years (range = 2 months – 89 years) | Native American | Non– American Indian/ Alaska Native population | January – December 2009 | 2009 Influenza A (H1N1) pandemic |
| Carville et al, 2007^4^ | Retrospective cohort | Australia (Western Australia) | Birth – 2 years | Aboriginal and/ or Torres Strait Islander | Non–Indigenous population | 1990 – 2000 | Seasonal influenza |
| CDC, 2009^5^ | Retrospective from surveillance data | USA (12 states**) | All age groups | American Indian/Alaska Native | Non–American Indian / Alaskan Native population | April – October 2009 | 2009 Influenza A (H1N1) pandemic |
| Chowell et al, 2012^6^ | Retrospective from surveillance data | USA (Maricopa County) | All age groups  Hospitalisations: Median 21 years (range = 0 – 96 years)  Deaths: Median 46 years (range = 0 – 80 years) | Native American | Non–Hispanic White population | April 2009 – April 2010 | 2009 Influenza A (H1N1) pandemic |
| Dee et al, 2010^7^ | Retrospective from surveillance data | New Zealand (Hutt Hospital) | Adults > 18 years  Median age 31 years (range = 15 – 81 years) | Maori | New Zealand Europeans | June – July 2009 | 2009 Influenza A (H1N1) pandemic |
| Dee et al, 2011^8^ | Retrospective from surveillance data | USA (10 states) | Hospitalisations: All age groups  Deaths: Children < 18 years | American Indian / Alaska Native (Non–Hispanic) | Non–Hispanic White population | April – August 2009 (First wave) & September 2009 – January 2010 (Second wave) | 2009 Influenza A (H1N1) pandemic |
| D’Onise et al, 2008^9^ | Retrospective from surveillance data | Australia (South Australia) | Children < 5 years | Aboriginal and/or Torres Strait Islander | Non–Indigenous population | 1996 – 2006 | Seasonal influenza |
| Fathima et al, 2018^10^ | Retrospective cohort | Australia (Western Australia) | Children ≤16 years | Aboriginal and/or Torres Strait Islander | Non–Indigenous population | 2000 – 2016 | Both seasonal and pandemic influenza |
| Flint et al, 2010^11^ | Retrospective from surveillance data | Australia (Top End, Northern Territory) | All age | Aboriginal and/or Torres Strait Islander | Non–Indigenous population | June – August 2009 | 2009 Influenza A (H1N1) pandemic |
| Foote et al, 2015^12^ | Retrospective cohort | USA (nation–wide) | Infant < 1 year | American Indian / Alaska Native | Total US population | April 2009 – March 2010 | 2009 Influenza A (H1N1) pandemic |
| Goggin et al, 2011^13^ | Retrospective from surveillance data | Australia (Western Australia) | All ages | Aboriginal and/or Torres Strait Islander | Non–Indigenous population | June – August 2009 | 2009 Influenza A (H1N1) pandemic |
| Gounder et al, 2014^14^ | Retrospective from surveillance data | USA (HIS Contract health service delivery area counties (Indigenous), 13 states (Benchmark)) | All ages | American Indian / Alaska Native | Total population in nominated states | 2001 – 2008 | Seasonal influenza |
| Groom et al, 2014^15^ | Retrospective from surveillance data | USA (HIS Contract health service delivery area counties) | All ages | American Indian / Alaska Native (non–Hispanic) | Non–Hispanic White population | January – December 2009 | 2009 Influenza A (H1N1) pandemic |
| Harris et al, 2010^16^ | Prospective case control study | Australia (North Queensland) | All ages  Hospitalisations: Median age 33 years | Aboriginal and/or Torres Strait Islander | Non–Indigenous population | May – August 2009 | 2009 Influenza A (H1N1) pandemic |
| Helferty et al, 2010^17^ | Retrospective from surveillance data | Canada (nation–wide) | All ages | Aboriginal (First Nations, Inuit, Métis) | Non–Indigenous population | April 2009 – April 2010 | 2009 Influenza A (H1N1) pandemic |
| Hennessy et al, 2016^18^ | Retrospective Case control study | USA (Alaska, Arizona, New Mexico, Oklahoma, Wyoming) | All ages | American Indian / Alaska Native | Non – American Indian / Alaska Native | April – December 2009 | 2009 Influenza A (H1N1) pandemic |
| Kelly et al, 2009^19^ | Retrospective from surveillance data | Australia (nation–wide) | All ages | Aboriginal and/or Torres Strait Islander | Non–Indigenous population | May – October 2009 | 2009 Influenza A (H1N1) pandemic |
| Lenzi et al, 2012^20^ | Retrospective from surveillance data | Brazil (Paraná) | All ages  Hospitalisations: Mean age 25·8 (range = 0–90 years) | Indigenous population | Non–Indigenous population | January – December 2009 | 2009 Influenza A (H1N1) pandemic |
| Li–Kim–Moy et al, 2016^21^ | Retrospective from surveillance data | Australia (Western Australia, Northern Territory) | All ages | Aboriginal and/or Torres Strait Islander | Non–Indigenous population | 2010 – 2013 | Both seasonal and pandemic influenza |
| Menzies et al, 2004^22^ | Retrospective from surveillance data | Australia (nation–wide) | All ages | Aboriginal and/or Torres Strait Islander | Non–Indigenous population | July 1999 – June 2002 | Seasonal influenza |
| Menzies et al, 2008^23^ | Retrospective from surveillance data | Australia (New South Wales, Northern Territory, Queensland, South Australia and Western Australia) | All ages | Aboriginal and/or Torres Strait Islander | Non–Indigenous population | July 2002 – June 2005 | Seasonal influenza |
| Mostaç o–Guidolin et al, 2013^24^ | Retrospective from surveillance data | Canada (Manitoba) | All ages | First Nations population | Non–First Nations population | First wave: May–August 2009  Second wave: October – January 2010 | 2009 Influenza A (H1N1) pandemic |
| Naidu et al, 2013^25^ | Retrospective from surveillance data | Australia (New South Wales, Northern Territory, Queensland, South Australia, Victoria, Western Australia) | All ages | Aboriginal and/or Torres Strait Islander | Non–Indigenous population | July 2005 – June 2010 | Both seasonal and pandemic influenza |
| NSW Public Health Network, 2009^26^ | Retrospective from surveillance data | Australia (New South Wales) | All ages | Aboriginal and/or Torres Strait Islander | General NSW population | May – August 2009 | 2009 Influenza A (H1N1) pandemic |
| Pennington et al, 2017^27^ | Retrospective from surveillance data | Australia (nation-wide) | All ages | Aboriginal and/or Torres Strait Islander | Non–Indigenous population | 2009 | 2009 Influenza A (H1N1) pandemic |
| Prasad et al, 2019^28^ | Prospective observational | New Zealand (Central, southern and eastern Auckland) | Women of reproductive age (15 - 45 years) | Maori | European or other ethnicities (excluding Maori, Pacific or Asian | 2013 – 2015 | Seasonal influenza |
| Prasad et al, 2020^29^ | Prospective observational | New Zealand (Kidz First Children’s Hospital, South Auckland) | < 1 year | Maori | European and other ethnicities (excluding Maori, Pacific or Asian) | Winter months (End of April to end of September) 2014 – 2016 | Seasonal influenza |
| Rolland–Harris et al, 2012^30^ | Retrospective from surveillance data | Canada (nation–wide excluding Ontario and Nova Scotia) | Women of reproductive age | Aboriginal (including Inuit, Metis, First Nations) | Non–Indigenous population | April 2009 – April 2010 | 2009 Influenza A (H1N1) pandemic |
| Rudge et al, 2010^31^ | Retrospective from surveillance data | Australia (New South Wales) | All ages | Aboriginal or Torres Strait Islander | Non–Indigenous population | April – August 2009 | 2009 Influenza A (H1N1) pandemic |
| Thomspon et al, 2011^32^ | Retrospective from surveillance data | USA (New Mexico) | All ages | American Indian | Non–Hispanic White population | September 2009 – January 2010 | 2009 Influenza A (H1N1) pandemic |
| Verrall et al, 2010^33^ | Prospective case–control (hospital–based) | New Zealand (Wellington, Hutt Valley) | All ages  Hospitalisations: mean age 26 years (range = 0–82 years) | Maori | Non–Maori (excluding Pacific Islanders) | June – August 2009 | 2009 Influenza A (H1N1) pandemic |
| Wenger et al, 2011^34^ | Retrospective from surveillance data | USA (Alaska) | All ages | Alaska Native | White population | September – October 2009 | 2009 Influenza A (H1N1) pandemic |
| Weinman et al, 2020^35^ | Retrospective from surveillance data | Australia (Northern Territory) | All ages | Aboriginal or Torres Strait Islander | Non–Indigenous population | 2007- 2016 | Both seasonal and pandemic influenza |
| Zarychanski et al, 2010^36^ | Cumulative case–control study (based on surveillance data) | Canada (Manitoba) | All ages | First Nations | Non–First Nations | April – Septermber 2009 | 2009 Influenza A (H1N1) pandemic |

# Table D: Notes about hospitalisation- and mortality-rate calculations

| **Study** | **Notes about calculations** |
| --- | --- |
| Brooks et al, 2012^3^ | Native American deaths 11/50 (p1513); benchmark deaths 39/50  Population denominator obtained from US census bureau 2010^37^ – total New Mexico population = 2,059179  Indigenous population at risk: 193,222  Benchmark population at risk: 1,865,957 |
| Carville et al, 2007^38^ | Table 2, p213:  Aboriginal children admitted = 107  Non-Aboriginal children admitted = 399  Results p 211: Live births 1990-2000 = 270,068 (6.4% = 17,366 were Aboriginal)  Non-Aboriginal = 252,702 |
| CDC, 2009^5^ | Population at risk obtained from the 2010 census^37^ for the 12 states included.  American Indian / American Native = 1,318,277  Non-American Indian/American Native = 41,627,773 |
| Dee et al, 2010^7^ | P46 – population at risk = 140, 000  Maori 16% = 22,400  European 67% = 93,800  Ethnicity of H1N1 inpatients (n=54): Maori 39% (=21), European 31% (=17) |
| Dee et al, 2011^8^ | Table 3 p627 provides population distribution and influenza-related hospitalisation rates  Table 5 p628 provides mortality rates and population distribution at risk |
| Fathima et al, 2018^10^ | Use of 2000-2012 figures (ie 13 year period= 156 months)  Aboriginal flu A HR = 32.5/100000 child years = 2.7 / 100 000 child months  Non Aboriginal flu A HR = 4.5/100 000 child years = 0.38 / 100 000 child months  Aboriginal flu B HR =10.3 / 100 000 child years = 0.86 / 100 000 child months  Non Aboriginal flu B HR 0.9 / 1000 000 child years = 0.08 / 100 000 child months  Denominators not provided for meta analysis – use of the birth cohort n for denominator (first line of results). I.e. 469 589 children born in WA between 1996 – 2012. 6.7% identified as being Aboriginal.  Flu A and B results added together:  Aboriginal HR = (78+24) / (469 589 x 0.067) = 102 / 31 462 = 0.00324201 = 2.1 per 100 000 person **months**  Non-Aboriginal HR = (150+30) / (469 589 x 0.933) = 180 / 438 127 = 0.00041084 = 0.3 per 100 000 person months |
| Goggin et al, 2011^13^ | Table 1 p 174 provides hospitalisation figures Indigenous vs non-Indigenous  2011 census^39^: Aboriginal and Torres Strait Islander population of WA = 69, 664  Non-Indigenous population of WA = 2,239,170-69,664 = 2,169,506 |
| Helferty et al, 2010^17^ | Population at risk derived from 2011 census^40^  Total Canadian population = 33, 476, 688  Aboriginal population = 1,400,685  Non-Aboriginal = 32,076,003 |
| Hennessy et al, 2016^18^ | Population at risk for the 5 states obtained from 2010 census^37^  Total Indigenous population = 929,645  Total population 13,476,404  Non-Indigenous population = 12,546,759 |
| Kelly et al, 2009^19^ | Table p 2 provides hospitalisation and death data and population at risk. However, total population used, and therefore benchmark population changed to non-Indigenous population: 21,373,998-534,350 = 20,839,648.  Non-Indigenous hospitalisation = 4833-803 = 4030  Non-Indigenous deaths = 186-24 = 162 |
| Lenzi et al, 2012^20^ | Indigenous hospitalisations = 30  Non-Indigenous hospitalisation = 1911 – 30 = 1881.  Population at risk from 2000 census^41^: Indigenous population of Parana = 31,488, Total population of Parana = 9,564,643, Non-Indigenous population of Parana = 9,533,155 (Benchmark population) |
| Menzies et al, 2004^22^ | Population at risk based on 2001 census figures: Indigenous population 410,003; Non-Indigenous population = (18,769,249 – 41,003) = 18,359,246 |
| Menzies et al, 2008^23^ | Population at risk based on 2001 census figures for NSW, NT, QLD, SA, WA  Indigenous population = 365,343  Non-Indigenous population = 13,025,113 |
| Mostaço–Guidolin et al, 2013^24^ | Population at risk based on 2011 census  Total population of Manitoba = 1,208,268  First Nations ethnicity = 0.072 x 1,208,268 = 86,995  Non-First Nations ethnicity = 1,121,273  From table 1 p e40: First wave (3-month period): First nations hospitalised= 114, Non-First nations hospitalised = 99. Second wave (3-month period) First Nations hospitalised =27, Non-First Nations hospitalised = 139. |
| Naidu et al, 2013^25^ | Hospitalisations : population at risk from 2006 census^42^ – NSW, NT,QLD,SA, Victoria, WA: Indigenous = 434,155; Non-Indigenous = 18,618,299  Deaths: population at risk from 2006 census^42^ – NSW, NT, QLD, SA, WA: Indigenous = 404,014; Non-Indigenous = 13,716,018 |
| NSW Public Health Network, 2009^26^ | Population at risk based on 2006 census (NSW): Indigenous population = 138,506; Non-Indigenous population = 6,410,671 |
| Pennington et al, 2017^27^ | Table 11 reports Indigenous HR = 125.5 / 100 000. Indigenous cases admitted to hospital = 807.  Therefore Indigenous population = 807 / 0.001255 = 643028  Non-indigenous HR = 20.3 / 100 000.  Non-indigenous cases admitted to hospital = 4 278.  Therefore non-indigenous population = 4278 / 0.000203 = 21 073 892  Mortality rate calculated in same manner |
| Prasad et al, 2019 | As per table 3:  Maori HR = (43 + 14) /(104 685 + 1360137) = 57 / 1464822 = 3.9 per 100 000 women-weeks = 16.9 per 100 000 women-months  European and other HR = (56 + 10) /(282 086 + 5 779 445) = 66 / 6 061 531 = 1.1 per 100 000 women-weeks = 4.7 per 100 000 women-months |
| Prasad et al, 2020 | Incidence rates provided, however adjusted for SES / not crude. Thereore for meta-analysis, population data provided in the paper state that the area has approx. 8500 infants < 1 year. 19% Maori, 27% European and other ethnicities. |
| Rolland–Harris et al, 2012^30^ | Table 5 p1323 provides total number of women of reproductive aged admitted to hospital from 12 April 2009 – 3 April 2010.  WRA = 991, of whom 151 are noted to be Inuit, Metis or First Nations. 835 are noted to be non-Aboriginal.  Using reference 27 – statistics Canada data from 2006 census^43^ – estimated total number of WRA in Canada (combine the 15-24 + 25-44 cohorts) = 6 548 525, of whom 281 975 identified as Aboriginal (4.3%).  Therefore, the Indigenous hospitalisation rate = 151 / 281 975 = 53.6 per 100 000 person-years = 4.5 per 100 000 person-months  Non-Indigenous HR = 835 / 6266535 = 13.3 per 100 000 person years = 1.1 per 100 000 person months.  HRR = 4.1 |
| Thompson et al, 2011^32^ | Population at risk from 2010 census^37^- Indigenous = 193,222; Non-Indigenous = 2,059,179 – 193,222 = 1,865,957 |
| Weinman et al, 2020^35^ | Crude figures provided by authors  Indigenous hospitalisations: 17, Indigenous population denominator: 699,525  Benchmark hospitalisations: 12, benchmark population denominator 1,632,095  Indigenous deaths:, Indigenous population denominator:  Benchmark deaths:, Benchmark population denominator: |
| Zarychanski et al, 2010^36^ | Population at risk based on population fraction of 0.072 from Mostaço-Guidolin et al^24^  From the 2011 census:  Total population of Manitoba = 1,208,268  First Nations ethnicity = 0.072 x 1,208,268 = 86,995  Non-First Nations ethnicity = 1,121,273 |

# Table E: Quality assessment of included studies (Adapted from JBI critical appraisal for cohort studies)^44^

Risk of bias:

| Low ✔ |
| --- |
| High ❌ |
| Unclear❓ |

| **Author, Year** | **Country** | **1. Were the two groups similar and recruited from the same population?** | **2. Were the exposures measured similarly to assign people to both exposed and unexposed groups?** | **3. Was the exposure measured in a valid and reliable way?** | **4. Were confounding factors identified?** | **5. Were strategies to deal with confounding factors stated?** | **6. Were the outcomes measured in a valid and reliable way?** | **7. Was appropriate statistical analysis used?** |
| --- | --- | --- | --- | --- | --- | --- | --- | --- |
| Baker et al, 2009^1^ | New Zealand | ✔ | ✔ | ✔ | ❓ | ❌ | ✔ | ✔ |
| Bandaranayake et al, 2011^2^ | New Zealand | ✔ | ✔ | ✔ | ✔ | ❌ | ✔ | ✔ |
| Brooks et al, 2012^3^ | US | ✔ | ✔ | ✔ | ✔ | ❌ | ✔ | ❓ |
| Carville et al, 2007^4^ | Australia | ✔ | ❓ | ❓ | ✔ | ❓ | ❓ | ✔ |
| CDC, 2009^5^ | US | ✔ | ✔ | ✔ | ✔ | ✔ | ✔ | ✔ |
| Chowell et al, 2012^6^ | US | ✔ | ✔ | ✔ | ✔ | ❓ | ✔ | ✔ |
| D'Onise et al, 2008^9^ | Australia | ✔ | ✔ | ✔ | ✔ | ❌ | ✔ | ❌ |
| Dee et al, 2010^7^ | New Zealand | ✔ | ✔ | ✔ | ✔ | ❌ | ✔ | ❓ |
| Dee et al, 2011^8^ | US | ✔ | ✔ | ✔ | ✔ | ❓ | ✔ | ❓ |
| Fathima et al, 2018^10^ | Australia | ✔ | ❓ | ❓ | ✔ | ❓ | ✔ | ✔ |
| Flint et al, 2010^11^ | Australia | ✔ | ✔ | ✔ | ✔ | ✔ | ✔ | ✔ |
| Foote et al, 2015^12^ | US | ❌ | ✔ | ✔ | ✔ | ❓ | ❓ | ❓ |
| Goggin et al, 2011^13^ | Australia | ✔ | ✔ | ✔ | ✔ | ✔ | ✔ | ✔ |
| Gounder et al, 2014^14^ | US | ✔ | ✔ | ✔ | ❓ | ❓ | ❓ | ❓ |
| Groom et al, 2014^15^ | US | ✔ | ✔ | ✔ | ✔ | ❓ | ❓ | ✔ |
| Harris, et al.,2010^16^ | Australia | ✔ | ✔ | ✔ | ✔ | ❓ | ✔ | ✔ |
| Helferty et al, 2010^17^ | Canada | ✔ | ❓ | ✔ | ❓ | ❌ | ✔ | ❓ |
| Hennessy et al, 2016^18^ | US | ✔ | ✔ | ✔ | ✔ | ✔ | ✔ | ✔ |
| Kelly et al, 2009^19^ | Australia | ✔ | ✔ | ✔ | ✔ | ❌ | ✔ | ✔ |
| Lenzi et al, 2012^20^ | Brazil | ✔ | ✔ | ✔ | ✔ | ✔ | ✔ | ✔ |
| Li-Kim-Moy et al, 2015^21^ | Australia | ✔ | ✔ | ❓ | ✔ | ❓ | ✔ | ✔ |
| Menzies et al, 2004 ^22^ | Australia | ✔ | ✔ | ✔ | ✔ | ❓ | ❓ | ✔ |
| Menzies et al, 2008^23^ | Australia | ✔ | ✔ | ✔ | ✔ | ❓ | ❓ | ✔ |
| Mostaço–Guidolin et al, 2013^24^ | Canada | ✔ | ✔ | ✔ | ✔ | ❓ | ✔ | ✔ |
| Naidu et al, 2013^25^ | Australia | ✔ | ✔ | ✔ | ✔ | ❓ | ❓ | ✔ |
| NSW Public Health Network, 2009^26^ | Australia | ✔ | ✔ | ❌ | ✔ | ❌ | ✔ | ✔ |
| Pennington et al, 2017^27^ | Australia | ✔ | ✔ | ❓ | ✔ | ❌ | ✔ | ✔ |
| Prasad et al, 2019^28^ | New Zealand | ✔ | ✔ | ✔ | ❓ | ❓ | ✔ | ✔ |
| Prasad et al, 2020^29^ | New Zealand | ✔ | ❓ | ❓ | ✔ | ✔ | ✔ | ❓ |
| Rolland–Harris et al, 2012^30^ | Canada | ✔ | ✔ | ✔ | ❓ | ❌ | ✔ | ❓ |
| Rudge et al, 2010^31^ | Australia | ✔ | ✔ | ✔ | ❓ | ❌ | ✔ | ✔ |
| Thomspon et al, 2011^32^ | US | ✔ | ✔ | ✔ | ✔ | ✔ | ✔ | ✔ |
| Verrall et al, 2010^33^ | New Zealand | ✔ | ✔ | ✔ | ✔ | ✔ | ✔ | ❓ |
| Weinman et al, 2020^35^ | Australia | ✔ | ✔ | ✔ | ✔ | ✔ | ✔ | ✔ |
| Wenger et al, 2011^34^ | US | ✔ | ✔ | ✔ | ✔ | ❓ | ✔ | ❓ |
| Zarychanski et al, 2010^36^ | Canada | ✔ | ✔ | ✔ | ✔ | ✔ | ✔ | ✔ |

# Table F: JBI^44^ risk of bias assessment - comments

Risk of bias:

| Low |
| --- |
| High |
| Unclear |

| **Author, Year** | **Country** | **1. Were the two groups similar and recruited from the same population?** | **2. Were the exposures measured similarly to assign people to both exposed and unexposed groups?** | **3. Was the exposure measured in a valid and reliable way?** | **4. Were confounding factors identified?** | **5. Were strategies to deal with confounding factors stated?** | **6. Were the outcomes measured in a valid and reliable way?** | **7. Was appropriate statistical analysis used?** |
| --- | --- | --- | --- | --- | --- | --- | --- | --- |
| Baker et al, 2009^1^ | New Zealand | Nationwide surveillance systems listed | Reliable measurements | Reliable measurements | Raw surveillance numbers - mentioned in discussion | Raw surveillance numbers | Laboratory confirmed | RR was calculated |
| Bandaranayake et al, 2011^2^ | New Zealand | Same population | Reliable measurements | Reliable measurements | Factors identified include - co-morbidities, unfavourable environmental factors, behavioural differences, differences in socio-cultural-economic status, differences in health service utilisation and increased genetic susceptibility | No strategies stated | Laboratory confirmed | RR was calculated |
| Brooks et al, 2012^3^ | US | New Mexico | Census data + hospital data | Census data + hospital data | Factors that have been suggested to account for this include remote rural locations and poverty resulting in delayed ⁄decreased access to healthcare, higher prevalence of underlying medical | No strategies stated | Laboratory confirmed | RR was not calculated |
| Carville et al, 2007^4^ | Australia | The *WADLS is an established and reliable data source | Maternal lineage indigeneity only, fails to account for paternal heritage | Maternal lineage indigeneity only, fails to account for paternal heritage | Factors identified include - pre-existing illnesses and vaccination history | Adjusted for geography | ICD codes without laboratory confirm | *RR was extracted |
| CDC, 2009^5^ | US | Same 12 states | Medical records+ population estimates | Bridging race method | Factors identified include - underlying chronic medical conditions and social determinants of health, should be topics for future investigation. | Adjusted by age, comorbidities in table 2 | Laboratory confirmed | RR was calculated |
| Chowell et al, 2012^6^ | US | Maricopa County -all hospitals | Medical records | Medical records | Factors identified include - admission delays, immunosuppression, cancer within the last 12 months, and chronic lung disease were identified | Risk of death among A/H1N1 inpatients adjusted by age, gender, ethnicity/race, pandemic wave, and antiviral treatment | Laboratory confirmed | RR was calculated |
| D'Onise et al, 2008^9^ | Australia | Hospital separation data included all private and public hospitals in South Australia | Using hospital records and census population data for denominators | Standard to document in hospital records + *ABS census data | Factors identified include - readmissions, vaccination | No strategies stated | Laboratory confirmed | No appropriate analysis |
| Dee et al, 2010^7^ | New Zealand | Same population | Reliable measurements | Reliable measurements | Confounding factors identified appropriately | No strategies stated | Laboratory confirmed | RR was not calculated |
| Dee et al, 2011^8^ | US | *BRFSS respondents | Self-report | Self-report | Factors identified include - certain underlying health conditions, differences in clinical management or self-care of underlying health conditions and reduced access to quality health care services, low prevalence of vaccination, lower income | Adjusted by age, seasons | Laboratory confirmed | Adequate information to calculate risk but not reported |
| Fathima et al, 2018^10^ | Australia | The WADLS is an established and reliable data source | Maternal lineage indigeneity only, fails to account for paternal heritage | Maternal lineage indigeneity only, fails to account for paternal heritage | Factors identified include - age, *PCV status | Adjusted for age and PCV status | Laboratory confirmed | IRR produced for aboriginal vs not |
| Flint et al, 2010^11^ | Australia | Admitted /emergency patients | Hospital admission records | Hospital admission records | Factors identified include - age, remote dwelling, smoke, alcohol, comorbidities, differential testing, over-crowding | Adjusted for age, remote dwelling, comorbidities | Laboratory confirmed | IRR was calculated |
| Foote et al, 2015^12^ | US | Alaskan hospitals vs general US | Indigenous status identified through the *IHS user population which includes all registered AI/AN people who received IHS-funded health care service at least once during the preceding 3 years | Indigenous status identified through the IHS user population | Factors identified include – changing hospitalization practices, improved socioeconomic conditions, community education and improved household and environmental factors | Adjusted by age and sex | ICD codes without laboratory confirm | No RR calculated but can calculate thereafter |
| Goggin et al, 2011^13^ | Australia | All West Australia residents if positive are notified | Via interview | Via interview | Factors identified include - age, gender, antiviral treatment, vaccination, comorbidities | Adjusted by stepwise multivariate logistic regression model | Laboratory confirmed | OR was calculated |
| Gounder et al, 2014^14^ | US | Excluded states without IHS | Indigenous status identified through the IHS user population | Indigenous status identified through the IHS user population | Factors identified include -socioeconomic and environmental risk factors, circulating RSV | Adjusted by age | ICD codes without laboratory confirm | No RR calculated |
| Groom et al, 2014^15^ | US | Limited to counties with IHS | Indigenous status identified through the IHS user population | Indigenous status identified through the IHS user population | Factors identified include - hurricane mortality etc | Adjusted by age | ICD codes | RR was calculated |
| Harris, et al.,2010^16^ | Australia | Townsville presentation | Self-report when entered into lab database | Self-report when entered into lab database | Factors identified include - age, comorbidities | 95% confidence interval; the model was adjusted for the confounding effects of age and ethnicity | Laboratory confirmed | OR was calculated |
| Helferty et al, 2010^17^ | Canada | Same population | The degree of completeness of data was a challenge for underlying conditions (57% complete) and Aboriginal origin (62% complete). | Aboriginal status was self-reported or recorded by a health care provider in most cases | Some mentioned underlying conditions but only 57% complete | No strategies stated | Laboratory confirmed | RR was not calculated |
| Hennessy et al, 2016^18^ | US | Same population | Medical records | Medical records | Confounding factors identified appropriately | Adequate adjustment | Laboratory confirmed | OR was calculated |
| Kelly et al, 2009^19^ | Australia | Australian government influenza surveillance reports + ABS data for denominator | ABS data | ABS estimate with corrections for under reporting for denominator and hospital data with status known | Factors identified include - gestational stage, comorbidities | Could not report age-stratified or age-adjusted rates or adjust for the presence of co-morbidities. | Laboratory confirmed | RR was calculated |
| Lenzi et al, 2012^20^ | Brazil | Same population | Reliable measurements | Reliable measurements | Confounding factors identified appropriately | Analysed by multivariable logistic regression. | Laboratory confirmed | OR was calculated |
| Li-Kim-Moy et al, 2015^21^ | Australia | State/territory wide data for Northern Territory and West Australia | Data collected from Indigeneity coding and >90% status available | Hospital coding data + but only projections for ABS data | Factors identified include - age, comorbidities, vaccinations | Adjusted for age | Laboratory confirmed | RR was calculated |
| Menzies et al, 2004 ^22^ | Australia | From multiple states | With regards to hospitalisation | With regards to hospitalisation | Factors identified include - age, crowded living conditions | Adjusted for age | ICD codes without laboratory confirm | RR was calculated |
| Menzies et al, 2008^23^ | Australia | From multiple states | Census data | Census data | Factors identified include - age, crowded living conditions | Adjusted for age | ICD codes without laboratory confirm | RR was calculated |
| Mostaço–Guidolin et al, 2013^24^ | Canada | Same population | Reliable measurements | Reliable measurements | Confounding factors identified appropriately | Adjusted by age | Laboratory confirmed | RR was calculated |
| Naidu et al, 2013^25^ | Australia | From multiple states | Census data | Census data | Factors identified include - age, crowded living conditions | Adjusted for age | ICD codes without laboratory confirm | RR was calculated |
| NSW Public Health Network, 2009^26^ | Australia | Same hospital catchments which account for 72% state | Same data sources | Other studies have shown New South Wales hospital records not reliable for indigeneity | Factors identified include – pregnancy, comorbidities, obesity | No strategies stated | Laboratory confirmed | RR was calculated |
| Pennington et al, 2017^27^ | Australia | National-wise study | With regards to hospitalisation | Projection from census data | Factors identified include - age, comorbidities | No strategies stated | Laboratory confirmed | RR was calculated |
| Prasad et al, 2019^28^ | New Zealand | Same population | Reliable measurements | Reliable measurements | Confounding factors identified incompletely. Stated 'vaccination rate' as a contributing factor but no mentioning of other confounding factors | Kept age and ethnicity for adjustment variable | Laboratory confirmed | RR was calculated |
| Prasad et al, 2020^29^ | New Zealand | South Auckland | Not explicitly stated. assuming obtained from hospital admin data | Not explicitly stated. assuming obtained from hospital admin data | Confounding factors identified appropriately | Comparisons were made after adjustment for SES | Laboratory confirmed | RR was not calculated |
| Rolland–Harris et al, 2012^30^ | Canada | Same population | Aboriginal status was self-reported by the patient and provided to the *PHAC, whenever available. | Aboriginal status was self-reported by the patient and provided to the PHAC, whenever available. | Factors identified include - age and underlying conditions | No strategies stated | Laboratory confirmed | RR was not calculated |
| Rudge et al, 2010^31^ | Australia | New South Wales net epi group | Sufficient rates of indigenous status | Sufficient rates of indigenous status | Risk factor data were incomplete - didn’t discuss seasonality | No strategies stated | Laboratory confirmed | RR was calculated |
| Thomspon et al, 2011^32^ | US | Same population | Reliable measurements | Reliable measurements | Factors identified include - age and obesity | Multivariate analyssis | Laboratory confirmed | RR was calculated |
| Verrall et al, 2010^33^ | New Zealand | Same population | Reliable measurements | Reliable measurements | Factors identified include - access to testing, local disease transmission dynamic | Age and comorbidities adjustment | Laboratory confirmed | RR was not calculated |
| Weinman et al, 2020^35^ | Australia | Northern Territory | Northern Territory indigeneity data has high response rates | Northern Territory indigeneity data has high response rates | Factors identified include age group, Indigenous status and area), seasonality of cases and spatial distribution of influenza types | Provided data relevant to all of the mentioned confounders | Laboratory confirmed | RR was calculated |
| Wenger et al, 2011^34^ | US | Same population | Reliable measurements | Reliable measurements | Factors identified include - underlying diseases, vaccination, age and race | Analysed in different groups | Laboratory confirmed | No RR but reasonable figures |
| Zarychanski et al, 2010^36^ | Canada | Same population | Reliable measurements | Reliable measurements | Confounding factors identified appropriately | Analysed by multivariable logistic regression. | Laboratory confirmed | OR was calculated |

*WADLS: West Australia Data Linkage System

*PCV: pneumococcal vaccine

*ABS: Australian Bureau of Statistics

*BRFSS: Behavioural Risk Factor Surveillance System
*HIS: Indian Health Service

*PHAC: Public Health Agency of Canada

# References

# List of Legends

S1 Text:

- Table A: Search Strategy
- Table B: List of excluded studies (from full text review)
- Table C: Features of included studies
- Table D: Notes about hospitalisation- and mortality-rate calculations
- Table E: Quality assessment of included studies
- Table F: JBI risk of bias assessment – comments
- References

S2: PRISMA Checklist
