# Supplementary material for: Influenza-associated hospitalisation and mortality rates among global Indigenous populations; a systematic review and meta-analysis
Source: PLOS Glob Public Health. 2023 Apr 13;3(4):e0001294. doi: 10.1371/journal.pgph.0001294 (PMC10101428; doi:10.1371/journal.pgph.0001294)
Supplement: Attachment — Submitted filename: Response to Reviewers.docx [file pgph.0001294.s003.docx]

18 December 2022

To the editors,

**Re: Influenza-associated hospitalisation and mortality rates among global Indigenous populations; a systematic review and meta-analysis – rebuttal letter**

Thank you for considering our manuscript. We are pleased to provide you with an updated copy of the manuscript which addresses the points below;

*1. Please provide separate figure files in .tif or .eps format*

Please find separate figure files in the requested .tif format.

*2. Please add a full list of legends for your Supporting Information files after the references list.*

Please find an updated version of the Supplementary Material which includes a full list of legends after the references list.

*3. Please add to the supplementary material: the searching strategy and the table of excluded articles (with the reason of exclusion)*

Please find an updated version of the Supplementary Material which now includes Table 1 – Search strategy and Table 2 – Excluded articles with reasons for exclusion.

*4. Check in line 176 if it is the right text “second reviewer”.*

This has now been changed to “verified by another author (AW/MN)”.

*5. Double check the title in the Table 2 (Supplementary Material)*

This has been changed to better reflect the contents of the table (now Table 4 in Supplementary material). The new heading reads, “Notes about hospitalisation- and mortality-rate calculations”.

*6. Draw PRISMA figure using the classic format, consolidating the final updated strategy, with the previous one.*

A new version of Figure 1 has been provided which conforms to the classic PRISMA format and consolidates the two searches.

Please find marked-up and “clean” copies of the manuscript uploaded to the editorial manager.

We look forward to hearing your response.

Yours Sincerely,

Dr Juliana Betts

on behalf of the authorship team
