# Supplementary material for: Influenza-associated hospitalisation and mortality rates among global Indigenous populations; a systematic review and meta-analysis
Source: PLOS Glob Public Health. 2023 Apr 13;3(4):e0001294. doi: 10.1371/journal.pgph.0001294 (PMC10101428; doi:10.1371/journal.pgph.0001294)
Supplement: Attachment — Submitted filename: Response to reviewers.docx [file pgph.0001294.s004.docx]

5 March 2023

To the editors,

**Re: “Influenza-associated hospitalisation and mortality rates among global Indigenous populations; a systematic review and meta-analysis”**

Thank you for sending our submission for review and for providing us with the opportunity to improve our manuscript. Please find below our responses to reviewer comments, all of which have now been addressed.

*Reviewer #1: The article raises a very important issue of health equity pertaining to the indigenous population. the manuscript however requires English editing to ensure usage of consistent and standard terminology throughout. Eg- "2009pH1N1" being used to denote the 2009 influenza pandemic. The manuscript would have been easier to review if the tables and figures were kept at the end of the main body instead of in between the body of the text.*

Response: Amendment made to ensure the use of “2009pH1N1” is consistent throughout the manuscript (see p. 22, line 376).

*Reviewer #2: This is a properly executed systematic review and meta-analysis on an important topic. I only have one comment (I am reviewing a revised version of the manuscript): country-level estimates for both USA and Canada rely solely on data from the 2009-10 H1N1 pandemic. This not only represents a limitation of the data, but also an important knowledge gap that needs to be addressed in future work. I think that this point warrants a sentence or two in the Discussion*

Response: The authors agree with this statement, and have added a paragraph to the discussion to reflect this point (see p. 22, line 383-389).

Journal Requirements:

*1. Please review your reference list to ensure that it is complete and correct. If you have cited papers that have been retracted, please include the rationale for doing so in the manuscript text, or remove these references and replace them with relevant current references. Any changes to the reference list should be mentioned in the rebuttal letter that accompanies your revised manuscript. If you need to cite a retracted article, indicate the article’s retracted status in the References list and also include a citation and full reference for the retraction notice.*

Response: The references are complete and correct.

*2. We have noticed that you have uploaded Supporting Information files, but you have not included a list of legends. Please add a full list of legends for your Supporting Information files after the references list.*

Response: Please find a list of legends at the end of the References list.

Thank you again for considering our work for publication.

Yours Sincerely,

Dr Juliana Betts, on behalf of the authorship team.

Research Fellow

School of Public Health and Preventive Medicine

Monash University

Melbourne, VIC, Australia

Phone: +61 407 235 433| Email: [juliana.betts@monash.edu](mailto:juliana.betts@monash.edu)
